# Clinical training alone is not sufficient for reducing barriers to IUD provision among private providers in Pakistan

**DOI:** 10.1186/1742-4755-8-40

**Published:** 2011-12-30

**Authors:** Sohail Agha, Aslam Fareed, Joseph Keating

**Affiliations:** 1 Population Services International, 1120 19th Street, NW, Suite 600, Washington, DC 20036, USA; 2 Greenstar Social Marketing, Technology Park, ST-8, Shara-e-Faisal, Karachi, Pakistan; 3 Tulane University SPHTM, 1440 Canal Street, New Orleans, LA 70112, USA

## Abstract

**Background:**

IUD uptake remains low in Pakistan, in spite of three major efforts to introduce the IUD since the 1960s, the most recent of these being through the private sector. This study examines barriers to IUD recommendation and provision among private providers in Pakistan.

**Methods:**

A facility-based survey was conducted among randomly selected private providers who were members of the Greenstar network and among similar providers located within 2 Kilometers. In total, 566 providers were interviewed in 54 districts of Pakistan.

Logistic regression analysis was conducted to determine whether correct knowledge regarding the IUD, self-confidence in being able to insert the IUD, attitudes towards suitability of candidates for the IUD and medical safety concerns were influenced by provider type (physician vs. Lady Health Visitor), whether the provider had received clinical training in IUD insertion in the last three years, membership of the Greenstar network and experience in IUD insertion. OLS regression was used to identify predictors of provider productivity (measured by IUD insertions conducted in the month before the survey).

**Results:**

Private providers consider women with children and in their peak reproductive years to be ideal candidates for the IUD. Women below age 19, above age 40 and nulliparous women are not considered suitable IUD candidates. Provider concerns about medical safety, side-effects and client satisfaction associated with the IUD are substantial. Providers' experience in terms of the number of IUDs inserted in their careers, appears to improve knowledge, self-confidence in the ability provide the IUD and to lower age-related attitudinal barriers towards IUD recommendation. Physicians have greater medical safety concerns about the IUD than Lady Health Visitors. Clinical training does not have a consistent positive effect on lowering barriers to IUD recommendation. Membership of the Greenstar network also has little effect on lowering these barriers. Providers' barriers to IUD recommendation significantly lower their monthly IUD insertions.

**Conclusions:**

Technical training interventions do not reduce providers' attitudinal barriers towards IUD provision. Formative research is needed to better understand reasons for the high levels of provider barriers to IUD provision. "Non-training" interventions should be designed to lower these barriers.

## Introduction

In spite of expert agreement that provider recommendations and practices can be extremely important in influencing the adoption of contraceptive methods [1-4], providers' attitudes, beliefs and practices related to contraceptive methods remain poorly understood. The few studies that have looked at provider attitudes and practices towards contraceptive provision have found that providers tend to impose restrictions related to client safety based on outdated medical information or based on moral concerns related to client behavior [5].

Provider barriers to the provision of contraceptive methods may be substantial in many developing country environments and are particularly important to identify in contexts such as Pakistan where contraceptive use has stagnated at a low level, 30% [6]. Moreover, the role of contraceptive methods in which the provider plays a role (such as the IUD, the injectable and the oral contraceptive) is declining, while the role of provider-independent methods (such as traditional methods or condoms) is increasing [7]. Over the last three decades, while a number of studies have looked at client-level determinants of contraceptive adoption in Pakistan [8-16], there is a dearth of published studies that have examined provider attitudes and practices towards the provision of contraceptive methods. One previous study that investigated the influence of the health service delivery environment did find several provider and health facility characteristics to be significant predictors of family planning adoption [17].

The present study looks at private providers' attitudes and practices related to the provision of the IUD. The IUD was first introduced into Pakistan's national family planning program in 1962, with 1,500 insertions being made by the end of 1963 [18]. Yet, in spite of its early introduction and the large number of insertions that occurred between 1965 and 1970, a much smaller number of women were using the IUD by the mid 1970s [19]. Renewed emphasis on the provision of the IUD through the public sector occurred during the 1980s with the introduction of the Copper-T IUD [20]. Yet, only 1.3% of currently married women of reproductive ages (MWRA) reported using the IUD in the 1990-91 Pakistan Demographic and Health Survey.

In 1995, social marketing of IUDs was initiated with the purpose of substantially expanding the supply of this method through the private sector. In a gender-segregated society such as Pakistan, women prefer to receive reproductive health services from health practitioners who are women. Independent, private, female physicians and paramedics (Lady Health Visitors or LHVs) were trained in counseling clients and provided clinical training in family planning service provision, including in IUD insertion and removal, by Greenstar Social Marketing, a Pakistani NGO supported by international donors [21]. These providers became part of a network of private providers called Greenstar whose membership offered quality assurance for clinical methods provision, subsidized contraceptives, and promotion of Greenstar-network clinics. The network was promoted initially through mass media [21] and subsequently through community based demand creation [22].

The introduction of the Multiload IUD through the private sector in 1995 was the third significant effort to introduce the IUD in Pakistan. Although the share of private sector provision of the IUD increased to nearly 50% by 2006-07 [6], up from 20% in 1990-91 [6], IUD use remained modest in Pakistan: the level of use of the IUD in 2006-07 (2.3%) was about the same as the level of use of the injectable (2.3%) or the oral contraceptive (2.1%) and was considerably below that of the condom (6.8%) or female sterilization (8.2%) [6]. Use of the IUD was also lower than the use of traditional methods such as rhythm (3.6%) or withdrawal (4.1%) [6]. Thus, the level of adoption of the IUD has remained much below early expectations of its role as a major contraceptive method that would reduce the birth rate in Pakistan [18,19].

In spite of multiple efforts to introduce the IUD in Pakistan, no previous publicly-available study has examined the role of provider attitudes and practices towards IUD provision in Pakistan. This study examines attitudes and practices of private providers in Pakistan, both members of the Greenstar network and non-members, towards recommending the IUD to reproductive age women. The findings reveal substantial provider barriers to the provision of the IUD and the effect of these barriers on IUD insertion. The findings are discussed in the context of the need for "non-training" interventions [23] targeting providers.

## Methods

The Tulane University Institutional Review Board (IRB) provided ethical approval for this study.

### Study design

Data were collected using a nationally representative sample of female private providers who are part of the Greenstar network and physicians and LHVs who provide the IUD but are not part of the Greenstar network. A list of 7,451 private providers who were members of the Greenstar network on December 31, 2008 was used as the sampling frame. Information on IUD sales by Greenstar medical representatives to these providers was available for all four quarters of 2008. Providers were stratified based on sales of IUDs in 2008. Providers who purchased the IUD during all four quarters of 2008 were classified as high-performers, providers who purchased the IUD during both halves of 2008 were classified as medium-performers, providers who purchased the IUD during either half of 2008 or did not purchase any IUD during 2008 were classified as low performers. High and medium performers were oversampled for this study in order to ensure that providers included in the study were active in IUD provision. Weights were created to adjust for oversampling of high and medium performers.

Because no updated sampling frame exists of private providers of the IUD who are not members of the Greenstar network, a mapping exercise was conducted in which all private providers of the IUD who were not members of the Greenstar network but were located within a 2 Kilometers radius of a Greenstar clinic were listed. This ensured that the sample frame of eligible female providers developed through the mapping exercise was similar on key characteristics such as geographic area and health facility catchment area, and inclusive with respect to eligible institutions. Simple random sampling was used to select private providers who were not members of the Greenstar network. No weights were attached to providers who were not members of the Greenstar network.

The survey was conducted in 54 out of 114 districts of Pakistan, excluding the Federally Administered Tribal Areas (7 districts) and the Federally Administered Northern Areas (17 districts). In total, 55 cities and 24 villages were visited by data collectors. About 95% of the sample of providers was urban, consistent with the footprint of the Greenstar network.

### Statistical analysis

All analyses were done using SPSS 14. Chi-square tests of independence were used to determine differences in the characteristics of members of the Greenstar network and other private providers (Table 1 in results section). Descriptive statistics were used to compare knowledge and attitudes towards the IUD among physicians vs. LHVs, providers who had received clinical training in the last three years vs. those who had not, current members of the Greenstar network vs. non-members and providers experienced in IUD insertion vs. those less experienced (Table 2 in results section). Adjusted odds ratios are presented to determine whether provider-type, clinical training, network membership and experience had independent effects on provider attitudes and barriers to IUD provision (Table 2).

**Table 1:** Greenstar network members' and other private providers' characteristics

	Member of the Greenstar network (n = 415)	Other private provider (n = 151)	P value
**Type of facility**			
Clinic	46.6	42.4	0.652
Hospital	25.6	28.5	
Maternity home	27.8	29.1	
			
**Type of provider**			
Doctor	53.0	48.3	0.092
Lady Health Visitor (LHV)	37.6	35.8	
Nurse/midwife/other	9.4	15.9	
			
**Trained in last 3 years in FP clinical skills**			
No	31.1	55.0	p < 0.001
Yes	68.9	45.0	
			
**Receive IUD supplies from Greenstar**			
No	8.7	35.8	p < 0.001
Yes	91.3	64.2	
			
**Insertions in career**			
< 45	18.3	25.8	p < 0.05
45 or more	81.7	74.2	
			
	100.0	100.0	

**Table 2:** Differences in knowledge and attitudes towards IUD provision among physicians versus LHVs, providers trained in last three years versus not, network members versus not, experienced providers (> 45 insertions) versus not

	Physician			Trained in clinical FP in last 3 years			Network member			Experienced provider		
	Yes n = 284	No n = 282	Adjusted^1 ^Odds Ratio	Yes n = 354	No n = 212	Adjusted Odds Ratio	Yes n = 415	No n = 151	Adjusted Odds Ratio	Yes n = 463	No n = 103	Adjusted Odds Ratio
**Knowledge of Multiload**	%	%		%	%		%	%		%	%	
Knows effectiveness	76.8	79.0		79.6	75.0		78.1	76.8		81.6	63.2	2.53***
Knows duration of effectiveness	76.1	74.0		77.9	70.4		75.9	72.8		76.5	69.6	
Has sufficient information to advise	93.9	96.0		98.3	89.6	3.42*	97.6	88.1	3.84**	98.2	82.6	8.26***
Considers herself expert at insertion	80.6	83.9		86.7	74.6		83.1	80.1		91.6	45.2	12.43***
**Appropriate candidates for IUD**												
If no contraindications, would recommend IUD to following women												
Nulliparous	11.2	20.6	0.48**	16.9	13.7		17.6	10.6	1.89*	16.2	13.9	
With one delivery	83.0	87.1		88.1	80.2	1.65*	86.5	80.8		85.6	83.5	
With 3+ deliveries	97.6	97.4		98.9	95.3		98.8	94.0	3.41*	98.7	93.0	4.63**
Age 19 or younger	34.1	50.2	0.48***	39.8	45.3	0.66*	42.7	39.7		42.0	40.9	
Age 20-24	90.4	93.4		93.8	88.7		92.5	90.1		93.8	84.2	2.48**
Age 25-29	97.3	94.9		98.3	92.0	4.51**	97.3	92.7		97.1	92.2	
Age 30-34	94.6	90.8		93.2	92.5		93.2	92.1		94.5	87.0	2.67**
Age 35-39	76.5	74.6		77.6	72.2		77.5	70.2		78.7	63.5	2.05**
Age 40 or older	46.3	43.4		44.9	44.6		43.9	47.7		50.6	22.6	3.80***
Wants children in future	85.7	87.2		87.3	85.0		87.0	85.4		87.6	82.5	
Had spontaneous abortion	31.7	37.9		35.7	32.9		34.2	35.8		35.7	30.4	
Had ectopic pregnancy	23.2	29.7	0.66*	22.9	31.9	0.55**	26.3	26.5		27.3	22.6	
The following concerns would negatively influence recommendation of the IUD												
Medical safety	79.6	63.6	2.30***	71.8	72.2		72.8	69.5		73.0	67.8	
Side-effects	71.4	64.3		69.8	65.1		68.9	65.6		68.4	66.7	
Client satisfaction	44.2	45.4		45.3	43.9		45.3	43.0		43.7	48.7	
Informed consent is cumbersome	20.8	17.6		22.9	13.2	2.47***	19.0	19.9		17.1	28.1	0.43**
Expense	4.4	3.3		4.0	3.8		3.9	4.0		2.7	8.8	0.25**

*p < 0.05 **p < 0.01 ***p < 0.001 ^1^Adjusted for other three independent variables in Table 2 (e.g. training in clinical FP provision, membership of network and experience in IUD provision)

Finally, Ordinary Least Squares (OLS) regression was used in a step-wise fashion to test whether provider type, clinical training during the last three years, network membership, experience in IUD insertion and provider attitudes towards and concerns about the medical safety of the IUD were associated with the number of IUD insertions in the last month. Two other variables, receipt of Greenstar supplies and type of facility were included in the OLS regression analysis but were dropped from the final model because they failed to achieve statistical significance. The probability of committing a type-1 error (alpha) was set at 0.05.

### Measures

All interviews were conducted at the health facilities at which the sampled providers worked. An instrument developed by Espey and colleagues (2003) was pretested and adapted for use in Pakistan. A 67-question survey instrument was used to collect data on basic information on the health facility and the providers, such as type of health facility (clinic, hospital, maternity home), provider qualification (doctor, LHV, other) and providers' knowledge, attitude, and practices related to the provision of the IUD. Training of interviewers was conducted between October and November 2009. Data were collected between November 2009 and November 2010.

#### Outcome variables

A broad range of outcome variables measuring knowledge, attitudes and practices were used for the analysis. Provider knowledge was assessed using questions related to the effectiveness and the duration of effectiveness of the IUD. Attitude measures were assessed using multiple questions related to providers' IUD-recommendation practices for clients of different ages, parity, fertility desires, and histories of spontaneous abortion or ectopic pregnancy. Providers were asked whether their concern for medical safety of the IUD, side effects, client satisfaction, and costs related to the IUD influenced their recommendation of the IUD. Providers' level of experience in IUD insertion was assessed by asking them about the number of IUD insertions they had performed during their career. Providers' provision of the IUD was measured by self-reports of IUD insertions performed in month before the survey.

#### Independent variables

For the analysis of factors associated with knowledge, attitudes and practices, the following independent variables were used: provider type was defined as a physician, LHV, or other; training was defined as whether the provider received clinical family planning training from any source in the last three years; membership of the Greenstar network was dichotomized as 1 if the provider was a member of the Greenstar network or 0 if not; the level of IUD experience was defined by whether the provider had inserted 45 or more IUDs during their career.

Several summary variables were created for the OLS regression analysis of factors associated with the number of IUDs inserted during the last month. A variable measuring medical safety/client satisfaction concerns was created as a simple count of the number of barriers to IUD insertion that were reported by providers. A variable measuring age/parity related barriers was created using a simple count of providers' willingness to recommend the IUD to women who were nulliparous, or women who were below age 20 or over age 40. A binary variable was created to identify providers who had correct knowledge of both the effectiveness and duration of effectiveness of the IUD. Binary variables were used to indicate whether providers considered women who had had a spontaneous abortion or women who had had an ectopic pregnancy as suitable candidates for the IUD.

## Results

### Characteristics of network and non-network providers

Table 1 shows characteristics of private providers who are members of the Greenstar network and of other private providers. There was no difference between members of the Greenstar network and non-members in terms of the type of facilities they were located in: more than 40% of providers worked in clinics, which are usually smaller facilities than hospitals or maternity homes. Nor was there any difference between members and non-members in terms of the type of provider: nearly half of all providers were physicians; a majority of the remaining providers were Lady Health Visitors (paramedics with 18 months of training in primary health care provision, including training in midwifery).

The proportion of providers who had received clinical training in IUD insertion during the last three years differed between members and non-members (69% vs. 45%, p < 0.001). More than 90% of both members and non-members who had received clinical training in the last three years had received this training from Greenstar (data not shown). Members of the network were also more likely to receive IUD supplies from Greenstar than non-members (91% vs. 64%, p < 0.001). Finally, our data show that network members were more experienced in IUD insertion than non-members (82% vs. 74%, p < 0.05).

### Knowledge

Table 2 shows knowledge about the effectiveness of the IUD and self-efficacy in advising clients about the IUD by provider type (physician versus Lady Health Visitor/other), clinical training during the last three years, membership of the Greenstar network and provider experience in IUD insertion.

Surprisingly, there was no significant difference between physicians and LHVs in terms of their IUD knowledge, nor was there a difference in their perception of being an expert in IUD insertion. Consistent with expectations, providers who had received clinical training during the last three years were more likely to feel that they had sufficient information to advise clients on the use of the IUD (98% versus 90%, AOR = 3.42, p < 0.05). However, providers' knowledge of IUD effectiveness or their perception of themselves as experts in IUD insertion was not influenced by having received clinical training in the last three years. A similar pattern was observed among network members: members of the Greenstar network were more likely to perceive themselves as having sufficient information to advise clients on IUD use (98% versus 88%, AOR = 3.84, p < 0.01) but their level of knowledge of IUD effectiveness or their self-confidence in being able to insert an IUD was not significantly different.

The only factor that had a significant effect on knowledge of IUD effectiveness was provider's greater experience with IUD insertion: 82% of providers who had inserted 45 or more IUDs in their career versus 63% who had inserted less than 45 IUDs in their careers knew the effectiveness of the Multiload IUD (AOR = 2.53, p < 0.001). Moreover, experienced providers also felt that they had sufficient information to advise on IUD use (98% versus 83%, AOR = 8.26, p < 0.001) and that they were experts at IUD insertion (92% versus 45%, AOR = 12.43, p < 0.001).

### Attitudes about appropriate candidates for the IUD

Physician and LHV attitudes regarding appropriate candidates for the IUD were very similar. Where attitudes differed, physician attitudes were more restrictive: physicians were less likely to consider nulliparous women suitable candidates for the IUD (11% versus 21%, AOR = 0.48, p < 0.01); physicians were less likely to consider women ages 19 and younger as suitable candidates (34% versus 50%, AOR = 0.48, p < 0.001); physicians were less likely to consider women who had ever had an ectopic pregnancy as suitable candidates (23% versus 30%, AOR = 0.66, p < 0.05).

Clinical training during the last three years had a mixed effect on provider attitudes towards candidates for the IUD: providers who had received clinical training in the last three years became more likely to consider women with one delivery and women ages 25-29 as appropriate candidates for the IUD; at the same time, these providers were less likely to consider women ages 19 and younger and women who had ever had an ectopic pregnancy appropriate candidates for the IUD.

Network membership did not have much influence on provider attitudes towards appropriate IUD candidates. Network membership was associated with provider attitudes in only two instances: network members were more likely to consider nulliparous women as appropriate candidates for the IUD (18% versus 11%, adjusted odds = 1.89, p < 0.05); network members were more likely to consider women who have had three deliveries as appropriate candidates (99% versus 94%, adjusted odds = 3.41, p < 0.05).

Greater experience in IUD insertion was consistently associated with less restrictive age-related barriers towards provision of the IUD: experienced providers were more likely to consider women ages 20-24, 30-34, 35-39 and 40 or above as suitable candidates for the IUD.

### Medical safety and client satisfaction concerns

Consistent with earlier findings from this study regarding their more restrictive attitude towards IUD provision, physicians were more likely to not recommend the IUD because of medical safety concerns (80% vs. 64%, AOR = 2.30, p < 0.001). A negative effect of clinical training on provider recommendation of the IUD was observed: clinical training lowered the providers' likelihood of recommending the IUD because it made providers feel that the informed consent procedures were too cumbersome (23% vs. 13%, AOR = 2.47, p < 0.001). Membership of the Greenstar network did not lower providers' concerns about the medical safety of the IUD.

Greater experience in IUD insertion had no influence on medical safety concerns. However, greater experience did lower other potential barriers: experienced providers were less likely to consider informed consent a barrier to IUD provision (17% vs. 28%, AOR = 0.43, p < 0.01); experienced providers were also significantly less likely to consider cost of the IUD to the client as a reason for not recommending the IUD (3% vs. 9%, AOR = 0.25, p < 0.01).

### IUD insertions performed

Table 3 shows factors associated with provider reports of the number of IUDs inserted by them in the last month. The table presents findings from OLS regressions. Variables were added in stages to determine whether the effects of variables in earlier models are explained by variables introduced in later models.

**Table 3:** Factors associated with the number of IUDs inserted in the last month (OLS regression)

	(1) Facility type + provider type + network member (n = 566)	(2) + network member (n = 566)	(3) + experience in IUD insertion (n = 566)	(4) + age/parity related barriers + medical safety concerns (n = 566)
**Constant**	3.50***	2.66***	-0.51	1.79
				
**Type of facility**				
Clinic	(r)	(r)	(r)	(r)
Hospital	1.65*	1.86*	1.75*	1.87**
Maternity home	0.09	-0.01	-0.01	0.30
				
**Type of provider**				
Doctor	(r)	(r)	(r)	(r)
LHV/other	2.38***	2.09**	1.82**	1.55**
				
**Network membership**				
No	(r)	(r)	(r)	(r)
Yes	1.52*	1.05	0.69	0.63
**Trained in last 3 years in FP clinical skills**				
No		(r)	(r)	(r)
Yes		2.02**	1.50*	1.73**
**Insertions in career**				
< 45			(r)	
45 or more			4.77***	4.50***
**Correct knowledge of IUD effectiveness/duration**				
Incorrect knowledge, either				(r)
Correct knowledge of both				0.07
				
**Age/parity-related barriers**				
Number of barriers				-0.52*
				
**Medical safety/client satisfaction concerns**				
Number of concerns				-0.57*
				
**Concern about history of spontaneous abortion**				
Would not recommend IUD				(r)
Would recommend IUD				-0.24
**Concern about history of ectopic pregnancy**				
Would not recommend IUD				(r)
Would recommend IUD				0.50
**R-squared**	4.0%	5.8%	12.6%	14.4%

*p < 0.05 **p < 0.01 ***p < 0.001

Type of facility, type of provider and membership of the Greenstar network are variables introduced in the first model. Hospitals conducted a significantly higher number of IUD insertions every month than clinics, possibly because of the larger size and greater volume of clientele at these facilities. There was no difference between maternity homes and clinics in terms of monthly IUD insertions. Lady Health Visitors inserted significantly more IUDs per month than physicians. Network membership was associated with higher monthly IUD insertions. Overall, the model explained 4% of the variance in the outcome, IUDs inserted during the last month.

Whether the provider received clinical training in the last three years is introduced next (Model 2, Table 3). Providers who had received clinical training in IUD insertion during the last three years conducted significantly more IUD insertions per month than providers who had not. Overall, the model explained 6% of the variance. Network membership did not have a significant independent effect on IUD insertions after the inclusion of clinical training in the model. This suggests that the effect of network membership on IUD insertion operates through clinical training. In other words, being part of the Greenstar network increases monthly IUD insertion because membership provides access to clinical training in IUD insertion.

Provider experience with IUD insertion is introduced in the third model. The level of experience that a provider has with IUD insertion is the strongest predictor of monthly IUD insertions: providers with more experience (those who had inserted 45 or more IUDs in their careers) conducted nearly five more IUD insertions per month than other providers, after controlling for other factors.

Variables included in the fourth and final model include correct knowledge regarding the IUD, age or parity-related related barriers, concerns about the medical safety of the IUD, provider willingness to recommend the IUD to women who had ever experienced a spontaneous abortion and provider willingness to recommend the IUD to women who had ever had an ectopic pregnancy. Provider's correct knowledge of the IUD's effectiveness, concern about a prior ectopic pregnancy or concern about a prior spontaneous abortion did not influence the provision of the IUD. However, the summary variable measuring age or parity-related barriers was significantly associated with IUD insertion, even after controlling for a range of other variables: each additional barrier was associated with one-half fewer IUD insertions per month. The summary variable measuring concerns about medical safety of the IUD or client's lack of satisfaction due to side effects of the IUD had a similar level of effect: each additional concern was associated with one-half fewer IUD insertions per month.

In the final model (Model 4, Table 3), several variables had an independent positive effect on IUD insertion: hospitals were associated with 1.9 more IUD insertions per month than clinics; Lady Health Visitors inserted 1.5 more IUD per month than physicians; clinical training in the last three years was associated with 1.7 more IUDs inserted per month; experienced providers inserted 4.5 more IUDs per month than less experienced providers. The full model explained 14% of the variance in the outcome.

## Discussion

Prior to this study, there was little in the way of systematic evidence about IUD-related knowledge, attitudes, and practices among private providers in Pakistan. This study has shown that while three-quarters of private providers have sufficient knowledge about the effectiveness of the IUD and consider themselves to be experts in IUD insertion, provider attitudes towards the suitability of candidates for the IUD leave considerable room for improvement. Essentially, providers consider women with one or more children or women in their peak reproductive years as ideal candidates for the IUD. Women ages 19 and below and women ages 40 and above are not considered appropriate candidates for the IUD. Moreover, very few providers consider nulliparous women as suitable candidates for the IUD. Across-the-board, providers have substantial concerns regarding the medical safety of the IUD, its side-effects and clients' lack of satisfaction with the method. These concerns are likely to be particularly important for private providers, since they rely on satisfied customers to ensure the financial viability of their practices.

Physicians have more restrictive attitudes towards provision of the IUD than Lady Health Visitors and are more concerned about the medical safety of the IUD. This finding suggests that greater effort needs to be made with physicians than with paramedics in order to lower provider barriers to the provision of the IUD. Lady Health Visitors are also significantly more likely to conduct a greater number of IUD insertions per month than physicians, even after controlling for their less restrictive attitudes towards client eligibility and fewer concerns about the medical safety of the IUD. On average, Lady Health Visitors insert 1.5 more IUDs per month than physicians. This is possibly because the service charge for the IUD (about $2.4) makes IUD provision more lucrative for Lady Health Visitors in comparison with the other primary care services they offer. Even though physicians charge more than Lady Health Visitors for the IUD ($3.8), these charges are low in comparison with the other services that they offer.

Clinical training was not associated in a consistent positive fashion with provider attitudes and beliefs regarding appropriate candidates for the IUD. This is an important finding which suggests that technical interventions which focus on increasing the capacity of providers to conduct certain procedures may fall short of changing provider attitudes and perceptions towards those procedures.

Membership of the Greenstar network did not have much influence on provider attitudes towards the suitability of candidates for the IUD. One instance in which membership of the network did have an effect was that members of the network were more likely to consider nulliparous women as suitable candidates for the IUD. Network membership did not influence medical safety concerns regarding the IUD. The lack of effect of network membership on provider barriers to IUD provision is understandable in that benefits of being a member of the network include technical training in IUD insertion, uninterrupted access to subsidized IUDs, and quality assurance visits by Greenstar's medical team. Yet, none of these benefits address provider barriers to recommendation of the IUD identified in this study. One noteworthy fact is that non-members also receive many of the benefits of network membership from Greenstar: Table 1 showed that nearly half of non-members also received clinical training in the last three years (compared to two-thirds of members); Table 1 also shows that two-thirds of non-members receive contraceptive supplies from Greenstar (compared to 91% of members). Because Greenstar provides services to both members of its network and other private providers, network membership may not be a significant differentiating factor between members and non-members.

The experience that a provider gains through the provision of the IUD seems to be the strongest determinant of improved knowledge of the IUD, greater confidence in being able to insert the IUD, and fewer restrictive attitudes towards its provision. These findings have major implications for the design of IUD training interventions in the private sector. The findings increase the importance of guided-learning experiences of IUD insertion, generating client demand for the IUD to ensure that providers get sufficient experience following training and the need for continued follow-up of providers to ensure that they conduct a minimum number of IUD insertions. More research may be needed to determine whether there is a particular minimum threshold level of experience of IUD insertion that is optimal for providers. One limitation of our study was in the way in which provider responses to the question regarding the number of IUDs inserted in their career were recorded: all providers who inserted more than 45 IUDs during their career were lumped together. This makes it impossible to determine a more precise relationship between how many IUDs a provider has inserted in her career and provider productivity (as measured by monthly IUD insertions).

The study findings show that medical safety concerns do not get lower as providers gain more experience in IUD insertion. This highlights the importance of conducting formative research to determine reasons for providers' medical safety concerns being so high in the case of the IUD. Another study, conducted in a very different setting, also found similar levels of medical safety concerns among providers of the IUD [2]. Once the reasons for medical safety concerns being so high are understood, "non-training interventions" may be designed to lower these concerns

The is perhaps one of the first studies to show a direct relationship between provider attitudes towards IUD insertion and provider behavior: the higher the number of age or parity-related barriers the less likely a provider is to insert an IUD; the higher the number of medical safety and client satisfaction concerns, the lower the likelihood of inserting an IUD.

The findings of this study emphasize the need to consider broader, non-training, approaches to lower barriers to the provision of the IUD among providers. Non-training interventions may include job aids, peer support, supportive supervision, client feedback and other approaches which have been found to have worked in improving provider, attitudes, beliefs and practices [23]. The findings suggest that, to be more successful in the provision of the IUD through the private sector, efforts to lower providers' attitudinal barriers will need to be stronger among providers with higher levels of formal medical training (i.e. among physicians). In order to improve the performance of private providers in IUD service delivery, more emphasis should be placed on directly addressing provider barriers to client eligibility and provider concerns about medical safety of this contraceptive method. A satisfied client is one of the most important priorities for private providers who rely completely reliant on their clientele for the survival of their business. Unless private providers are convinced that client dissatisfaction with the method will not lead to a loss of clientele, they are likely to remain reluctant to recommend the IUD.

Independent of the type of provider, providers based in hospitals insert 1.9 more IUDs per month than providers based in smaller clinics. This may, in part, reflect the higher demand for IUDs at hospitals compared to clinics. It is possible that clients perceive private hospitals as providing services at a higher level of quality than smaller private clinics and prefer to go to hospitals for procedures.

There was no independent effect of network membership on monthly IUD insertions. The effect of network membership operated through clinical training: on average, providers who had received clinical training inserted 1.7 more IUDs per month than other providers. Thus, while it did not consistently reduce attitudinal barriers to IUD insertion, clinical training was associated with increased provider productivity.

In the regression analysis, 86% of the variance associated with the number of insertions performed remains unaccounted for suggesting that client-level attitudes, beliefs and behavioral factors may play an important role in determining provider productivity. A recent study found that perceived support from their mothers-in-law, belief in the benefits of spacing, and the expectation of getting good quality services are important determinants of a Pakistani woman's intentions to adopt the IUD [8].

This study has several methodological limitations. One limitation has already been mentioned: provider responses to the question on the number of IUDs they had inserted in their career were lumped together for those providers who had inserted 45 or more IUDs. A second limitation is that the study relies on provider self-reports of IUD insertions performed. As with all self-reported information, the extent to which self-reports reflect actual behavior is not known. Provider self-reports of IUD insertions performed were, however, internally consistent over time: self-reported data on IUD insertions from this survey was consistent with self-reported IUD insertion data collected from the same providers during a census of Greenstar providers in May-June 2010. Because of the lack of systematic record-keeping at private provider clinics in Pakistan, it was not possible to cross-check the reliability of self-reported IUD insertion data against clinic statistics.

Non-training interventions designed to lower provider barriers to IUD insertion could initially be targeted towards the most experienced members of the Greenstar network (i.e. those who have inserted 45 or more IUDs in their career), providers based in hospitals and Lady Health Visitors. Targeting experienced and more productive providers who are based in facilities with higher demand for the IUD procedure is likely to be a more productive programmatic strategy with visible positive effects on increasing the acceptability and adoption of the IUD among Pakistani women. Moreover, prior to conducting formative research and designing more effective non-training interventions the discussion of the medical safety of the IUD could be made a part of the quarterly quality assurance visits made by Greenstar's physicians to members of the Greenstar network and the detailing visits by medical representatives employed by Greenstar to maintain regular contraceptive method supply. Such approaches could be an integral part of efforts to make provider attitudes towards the suitability of clients for the IUD less restrictive and to lower their medical safety and client satisfaction concerns.

## Conclusions

The study findings highlight the need to better understand provider attitudes and perceptions towards the IUD. Providers' attitudinal barriers to IUD recommendation, related to providers' perceptions of the appropriateness of the IUD for certain types of clients or to their concerns about client safety, substantially reduce the number of IUDs inserted by trained providers. Clinical training is unable to address provider attitudinal barriers to IUD provision. Interventions that address "non-training" elements are needed to increase provider productivity in terms of IUD insertions. Networks that aim to increase the provision of contraceptive methods through technical training of private providers should implement effective approaches to lowering attitudinal barriers to method provision.

## Competing interests

SA is an employee of Population Services International and served as Senior Technical Advisor to Greenstar Social Marketing between October 2008 and December 2011. AF is an employee of Greenstar Social Marketing.

## Authors' contributions

SA was responsible for the design of the study, the data analysis and for the write-up of the report. AF was responsible for developing the sampling frame, supervising the study and assisting in the data analysis. JK assisted with the write-up of the study and the interpretation of study findings. All authors read and approved the final manuscript.
